# Assessing organizational readiness for depression care quality improvement: relative commitment and implementation capability

**DOI:** 10.1186/s13012-014-0173-1

**Published:** 2014-12-02

**Authors:** Lisa V Rubenstein, Marjorie S Danz, A Lauren Crain, Russell E Glasgow, Robin R Whitebird, Leif I Solberg

**Affiliations:** RAND Corporation, 1776 Main Street, Santa Monica, CA 90401 USA; Veterans Affairs Greater Los Angeles Healthcare System, North Hills, CA 91343 USA; HealthPartners Research Foundation, Minneapolis, MN 55440 USA; Division of Cancer Control and Population Sciences, National Cancer Institute, Rockville, MD 20852 USA

**Keywords:** Depression, Primary care, Quality improvement, Readiness, Measurement

## Abstract

**Background:**

Depression is a major cause of morbidity and cost in primary care patient populations. Successful depression improvement models, however, are complex. Based on organizational readiness theory, a practice’s commitment to change and its capability to carry out the change are both important predictors of initiating improvement. We empirically explored the links between relative commitment (i.e., the intention to move forward within the following year) and implementation capability.

**Methods:**

The DIAMOND initiative administered organizational surveys to medical and quality improvement leaders from each of 83 primary care practices in Minnesota. Surveys preceded initiation of activities directed at implementation of a collaborative care model for improving depression care. To assess implementation capability, we developed composites of survey items for five types of organizational factors postulated to be collaborative care barriers and facilitators. To assess relative commitment for each practice, we averaged leader ratings on an identical survey question assessing practice priorities. We used multivariable regression analyses to assess the extent to which implementation capability predicted relative commitment. We explored whether relative commitment or implementation capability measures were associated with earlier initiation of DIAMOND improvements.

**Results:**

All five implementation capability measures independently predicted practice leaders’ relative commitment to improving depression care in the following year. These included the following: quality improvement culture and attitudes (*p* = 0.003), depression culture and attitudes (*p* <0.001), prior depression quality improvement activities (*p* <0.001), advanced access and tracking capabilities (*p* = 0.03), and depression collaborative care features in place (*p* = 0.03). Higher relative commitment (*p* = 0.002) and prior depression quality improvement activities appeared to be associated with earlier participation in the DIAMOND initiative.

**Conclusions:**

The study supports the concept of organizational readiness to improve quality of care and the use of practice leader surveys to assess it. Practice leaders’ relative commitment to depression care improvement may be a useful measure of the likelihood that a practice is ready to initiate evidence-based depression care changes. A comprehensive organizational assessment of implementation capability for depression care improvement may identify specific barriers or facilitators to readiness that require targeted attention from implementers.

**Electronic supplementary material:**

The online version of this article (doi:10.1186/s13012-014-0173-1) contains supplementary material, which is available to authorized users.

## Background

Depression is a major cause of morbidity and cost in primary care patient populations [1]. Yet improving depression care is complex, requiring attention to all aspects of the chronic care model [2,3]. The highly evidence-based, multicomponent depression collaborative care model, for example, focuses on trained care managers, enhanced mental health specialty/primary care collaboration, and patient self-management support [4,5]. This model may require between 3 months and a year to implement, even with leadership commitment and availability of tools and assistance [6]. Simpler models, such as education [7] or clinical reminders [8,9] alone, have not improved depression outcomes. Overall results for implementation of collaborative care, however, have often masked substantial variations in model initiation, implementation, and outcomes across participating primary care practices [6,10,11] and providers [12]. In this paper, we empirically investigate key concepts from the organizational readiness for change literature [13,14] as an approach for understanding variations in initiating depression care improvement, the necessary first step for successful collaborative care implementation.

Organizational readiness theory suggests that contextual factors (i.e., the key features of the environment surrounding the improvement) may predict readiness to improve care and explain variations in uptake. Some analysts suggest that lack of organizational readiness for change may account for as many as half of all unsuccessful change initiatives resulting in complex redesign efforts [15,16]. Yet there is little empirical validation of the multiple methods available for assessing readiness [13,16-19]. In addition, progress toward empirical validation of readiness measures has been hampered by lack of a common theoretical or conceptual understanding of readiness in quality improvement programs [20,21].

Based on extensive review of prior literature, Weiner [13] postulated that both change commitment (a psychological construct) and change efficacy (a measure of perceived capability for the desired change) determine readiness. These in turn are shaped by how favorably members assess implementation capability, or the task demands, resource availability, and situational factors likely to act as barriers or facilitators for change.

Most current measures of readiness are not conceptualized in terms of transient readiness states, although evidence suggests that readiness for collaborative care can change over time [11]. The transtheoretical model (TTM) of readiness for change with its conceptualization of progressive stages of change [14,22] has proven to be highly useful for predicting and working with habit change in individuals. This model conceptualizes five stages of change (pre-contemplation, contemplation, preparation, action, and maintenance). In the pre-contemplation stage, individuals begin to learn about changes. In the contemplation stage, pros and cons are being weighed and changes in the near future are being considered. In the preparation stage, individuals are ready to make changes in the near future. In the action stage, some changes have been made. And in the maintenance stage, changes have been made and a period of time has passed. This framework may be applicable to understanding organizational change as well [23]. We conceptualize the development of change commitment as a precursor to action. Practices that show low change commitment may benefit from collaborative care preparation approaches (e.g., education, analysis of local data) aimed at promoting commitment rather than action; evaluations may learn more by accounting for low readiness.

In the exploratory work presented here, we ask whether primary care practice leaders’ intention to initiate change (measured here as *relative commitment* to depression care improvement versus to other potential practice improvement priorities in the following year) is associated with a set of specific contextual features chosen to reflect the practice’s *implementation capability*. We also explore whether these factors are related to the practice’s early participation in implementing depression care improvements.

## Methods

### Setting

We base our investigation on data from the Depression Improvement Across Minnesota, Offering a New Direction (DIAMOND) initiative, a quality improvement project for implementing depression collaborative care [24-27]. The goal of the DIAMOND initiative is to assist regional medical groups in Minnesota that belong to the Institute for Clinical Systems Improvement (ICSI) in implementing evidence-based collaborative care for depression. ICSI is a non-profit health care improvement organization that includes over 45 medical groups. The DIAMOND study will evaluate the effect of change in reimbursement and facilitated organizational change on the success of implementation and outcomes. ICSI recruited practices for participation in the initiative, and the study team recruited for participation in the evaluation. All practices participating in the initiative agreed to participate in the evaluation.

### Data collection

We used organizational context survey data collected, as part of the DIAMOND study, from February to April 2008 from each of the 83 practices. In all cases, the survey data were collected prior to implementation; however, the time between survey data collection and initiation of implementation varied among the different practices because the timing of implementation varied (see *Timing of Implementation*). We also used demographic data on practice location and staffing and demographic data on medical group size, mental health specialist availability, reimbursement, and ownership collected from the regional medical groups to which the practices belonged.

#### Survey data collection

We evaluated the data collected from two simultaneously administered practice surveys to measure relative commitment to depression care improvement and its associated features—the Physician Practice Connection Questionnaire (modified to focus on depression care) and the Change Process Capability Questionnaire [26,28]. Items in the questionnaires generally had three to five response options. For example, the item ‘*Does your clinic*(*s*) *have a system to identify and send reminders to patients who are due for the following services*? *Renewal for antidepressants*’ has the following response options: ‘*Yes*, *works well*; *Yes*, *needs improvement*; *No*; *Do not know*’. Each practice’s medical leader (usually a physician) completed the Physician Practice Connection Questionnaire. Each practice’s quality improvement leader (usually the clinic manager) completed the Change Process Capability Questionnaire. The response rates were very high—99% (82/83) for the Physician Practice Connection Questionnaire and 100% (83/83) for the Change Process Capability Questionnaire.

### Measures

**Figure 1:**
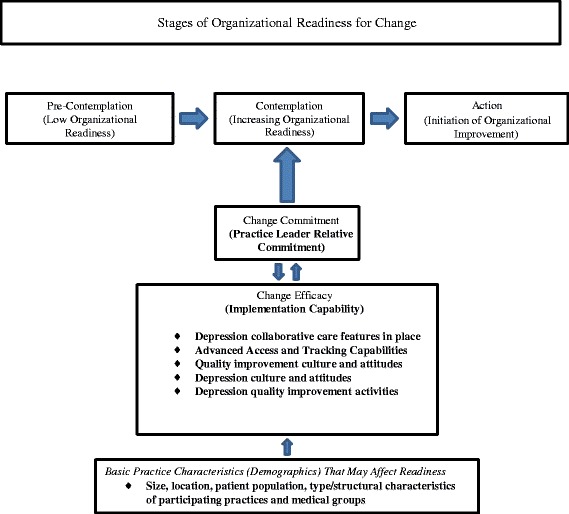
**Predicting organizational readiness for depression care improvement (measured composites in bold).**

Our conceptual and analytic framework for measurement is shown in Figure 1. Our primary dependent variable is practice leaders’ relative commitment to depression care improvement over the following year based on the priority expressed by the practice leaders. We tested the implementation capability measures as predictors of relative commitment, as well as practice and medical group demographic characteristics, all measured at the time of enrollment in DIAMOND. Finally, we explore whether either relative commitment or implementation capability predicts timing of initiation of DIAMOND depression care improvements, measured as the date, among available options when a practice started its DIAMOND implementation (termed implementation wave).

#### Practice leaders’ relative commitment

The practice leaders’ relative commitment to depression care improvement is based on averaging ratings for each practice’s medical leader and clinic manager/quality improvement coordinator. These leaders independently rated their practice’s relative priority for improving depression care over the following year on the same question: ‘On a scale of 1–10 where 1 = not a priority and 10 = highest priority of all and considering all the priorities your clinic has over the next year (e.g., EMR, financial goals, quality improvement of various conditions, MD recruitment), what is the priority of your clinic for improving depression care?’ By averaging the ratings, we aimed to integrate the relative commitment for the two different perspectives within the practice. Overall, the mean score for relative commitment for medical leaders was 5.8 on the 10-point scale (SD = 2.3, *n* = 81) and that for the clinic manager/quality improvement coordinators was 6.5 (SD =1.8, *n* = 78).

#### Medical group demographic characteristics

Medical group here refers to an association of physicians (including primary care providers, specialists, and other health care providers) which contracts with a payer to provide services to enrollees. Measured demographics include the following: numbers of primary care practice sites, psychiatrists, and mental health therapists; ownership; and payer mix.

#### Primary care practice demographic characteristics

We assessed staffing (numbers of physicians, nurse practitioners, physician assistants, and registered nurses).

#### Implementation capability

While the survey questions had been previously developed largely in relationship to the Chronic Illness Care Model for the purposes of measuring organizational readiness, we used expert opinion to more closely map questions to readiness concepts (e.g., experience with task demands such as prior implementation of depression collaborative care features, availability of resources such as patient tracking capabilities, and situational factors such as quality improvement culture [13]). We saw these concepts as also linking to other prior theory related to innovation adoption (e.g., values, trialability, and relative advantage [29]). To develop the composites, two of the authors (LR and MD) independently used clinical judgment to place survey items in categories. Differences in assignment were discussed and resolved. This process produced five composites as shown in Figure 1: 1) advanced access and tracking capabilities, 2) depression collaborative care features in place, 3) quality improvement culture and attitudes, 4) depression culture and attitudes, and 5) prior depression quality improvement activities (see ‘Examples of items in implementation capability composites’ below and the full set of questions in Additional file 1). Scores for each composite reflect the degree to which the items in a composite described a given practice at the time of the surveys, prior to implementing DIAMOND. Higher scores suggest more favorable context for program participation.

Examples of items in implementation capability composites:

Depression collaborative care features in place–*reflects having care management, review of non-improving cases by a psychiatrist, and systems for identifying depressed patients*:

- ♦ Routine components of care management that are provided to patients with depression,
- ♦ Activities to encourage patient self-management,
- ♦ Responsibilities of non-physician staff members in the care of depression patients,
- ♦ Presence of a care manager to provide education and follow-up.

Advanced access and tracking capabilities–*reflects system support for continuity and same-day scheduling, problem lists, medication lists, and prevention alerts or reminders*:

- ♦ Electronic and paper patient tracking tools,
- ♦ Patient reminders for medication refills and preventive services,
- ♦ Provider reminders regarding status of age-appropriate preventive services.

Quality improvement culture and attitudes–*reflects whether the practice operations relied heavily on organized systems, had systems-oriented leadership and clinicians with quality improvement skills, and had a shared mission*:

- ♦ Operations rely heavily on organized systems,
- ♦ Well-developed administrative structures and processes in place to create change,
- ♦ Well-defined quality improvement process for designing and introducing changes in the quality of care,
- ♦ Agreement by clinicians to follow evidence-based treatment guidelines for screening tests, immunizations, risk assessments, and counseling.

Depression culture and attitudes–*reflects the degree to which individual clinicians and practice leaders think they should improve care for depression and follow guidelines for it*:

- ♦ Agreement by clinicians to follow evidence-based treatment guidelines for depression and preventive services,
- ♦ Belief by clinicians that good depression care is very important,
- ♦ Leadership strongly committed to the need for change and to leading that change in depression care.

Prior depression quality improvement activities–*reflects the extent to which the practice already has identified depression improvement champions and teams, is looking at depression performance measures, is tracking depressed patients, and is undertaking measurement-based improvements*:

- ♦ Strategies to implement improved depression care (e.g., skills training, opinion leaders to encourage support for changes, measures to assess compliance and performance against goals, iterative approach to introducing changes, registry of patients to monitor programs, and track follow-up needs).

#### Timing of initiation of the DIAMOND depression care improvements

We documented the DIAMOND implementation date for each practice [25]. The practices that agreed to participate began implementation in five waves. These waves occurred every 6 months over a 2-year period between 2008 and 2010. Wave assignment reflected both practice willingness to begin at a given time and direction from ICSI. ICSI assigned practices to different waves, or sequences, based, in part, on the DIAMOND leadership efforts to balance the number and distribution of practices for each wave. For the earlier waves in particular, which required initiation of implementation with less advanced warning, ICSI engaged practices perceived as having high willingness or ability to participate. In some cases, for example, a practice scheduled to begin in one wave was moved to a later one because the original start date could not be met. For a given wave, training by the DIAMOND initiative took place over 6 months and implementation started just after, as the next wave began training. At the time of the survey, *wave 1* was between 6 and 7 months away from implementation and some respondents may have begun their training. No other wave had started training, and the training was 24 months in the future for the last wave, *wave 5*. Some practices, initially intending to join, never implemented the program (indicated as ‘none’). For the analyses, the non-implementing practices were treated as a separate group with a value of ‘6’ as if they were in a later sequential group than 5.

### Data analysis

#### Measures

We calculated a score for each implementation capability composite for each practice. The score for each item within a composite ranged from 0 to 1. The score for items with three response options became 0/0.5/1; four response options became 0/0.33/0.67/1, etc. Higher values reflected greater facilitators and/or fewer barriers to implementation capability. Each implementation capability composite score was calculated by averaging the item scores so that the result could be interpreted as reflecting the proportion and degree to which items were in place to facilitate implementation in each practice. We used Cronbach’s alpha to assess the degree to which items in the composites were rated in the same way (i.e., represented the same theoretical construct). We used the Pearson correlation coefficient to assess collinearity among the five composites.

#### Descriptive and bivariate analyses

Measures of central tendency and dispersion were calculated for each measure included in the analyses in a manner appropriate to its scale. We used general linear regression to predict practice leaders’ relative commitment to improvement from each implementation capability composite, as well as from practice or medical group demographic characteristics. Values that represented the most populous or the normative condition as indicated by the measures of central tendency for a demographic characteristic, or that represented the absence of the characteristic being measured, were selected as reference values. We also assessed the degree to which each implementation capability composite independently accounted for variance in the practice leaders’ relative commitment score using the *R*^2^ statistic. To ease the interpretation, we calculated model-predicted practice leaders’ relative commitment scores to correspond to hypothetical practices with high (75th percentile), medium (50th percentile), or low (25th percentile) implementation capability scores.

We also used GLM to estimate the relationships between implementation wave and practice/medical group demographics, practice leaders’ relative commitment to improvement, and the implementation capability composites. Models predicting timing of initiation of the DIAMOND improvements were also estimated using ordinal logistic regression, an approach that is more appropriate for modeling ordinal outcomes that are non-normally distributed such as categorical or count data. In none of the model pairs did the results of the GLM offer an interpretation that differed from that of the ordinal logistic model. For ease of explanation, the simpler GLM results are presented.

## Results

### Practice demographic characteristics

Among the primary care practices participating in the DIAMOND initiative, just over half of the 83 included in this dataset (57%) were from the Twin Cities metropolitan area. The practices were mostly medium-sized, with approximately two-thirds (65%) having 3–10 adult primary care physicians; 22% were large with more than 10 adult primary care physicians. Most practices were adult primary care only; 60% had no specialists. Most practices, however, included nurse practitioners or physician assistants (76%); 19% had no registered nurses.

### Characteristics of the medical groups to which practices belonged

The majority of practices (59%) belonged to medium-sized to large medical groups with more than ten primary care sites. Most groups had associated mental health support; 59% of practices belonged to a medical group with at least one associated psychiatrist and 60% belonged to a group with at least one mental health therapist. In 63% of the practices, over 50% of the patients had commercial insurance, but a substantial proportion of patients had only government insurance. In 40% of the practices, 25%–50% of the patients were insured through Medicare; and in half of the practices, more than 10% of patients were insured through Medicaid. Most medical groups (66%) were owned by hospital or health systems; almost a third (29%), however, were physician-owned.

### Practice implementation capability

The five implementation capability composites had internal reliabilities (raw Cronbach’s alpha) as follows: advanced access and tracking capabilities (*α* = 0.79; 18 items), depression collaborative care features in place (*α* = 0.90; 29 items), depression culture and attitudes (*α* = 0.55; 5 items), prior depression quality improvement activities (*α* = 0.88; 25 items), and quality improvement culture and attitudes (*α* = 0.78; 14 items).

The practices scored highest on two implementation capability composites: quality improvement culture and attitudes (*M* = 0.73, SD = 0.16, *n* = 83) and depression culture and attitudes (*M* = 0.70, SD = 0.19, *n* = 83). Prior depression quality improvement activities (*M* = 0.32, SD = 0.18, *n* = 83) and depression collaborative care features in place (*M* = 0.25, SD = 0.16, *n* = 83) scored lowest. Advanced access and tracking capabilities (*M* = 0.57, SD = 0.16, *n* = 82) had a score in the middle range.

Correlations among the five implementation capability composites were significant and moderate to high. For the composite labeled depression collaborative care features in place, correlations ranged from 0.29 (quality improvement culture and attitudes) to 0.42 (prior depression quality improvement activities). For advanced access and tracking capabilities, the range was from 0.22 (prior depression quality improvement activities) to 0.42 (quality improvement culture and attitudes). For depression culture and attitudes, the range was from 0.33 (advanced access and tracking capabilities) to 0.68 (quality improvement culture and attitudes). For prior depression quality improvement activities, the range was from 0.22 (advanced access and tracking capabilities) to 0.54 (depression culture and attitudes). For quality improvement culture and attitudes, the range was from 0.29 (depression collaborative care features in place) to 0.68 (depression culture and attitudes).

### Describing and predicting practice leaders’ relative commitment to depression improvement

The mean score for practice leaders’ relative commitment across all practices was 6.1 on the 10-point scale (SD = 1.7, *n* = 83). For all implementation capability composites, greater implementation capability was significantly associated with higher practice leaders’ relative commitment to improvement. The composites for depression culture and attitudes (*R*^2^ = 0.18) and prior depression quality improvement activities (*R*^2^ = 0.19) were most strongly associated with relative commitment (*p* < 0.001) and accounted for the largest proportions of its variance.

Only two of the medical group and primary care practice demographic characteristics we measured were significantly related to practice leaders’ relative commitment scores. We found higher scores for practices located in an urban setting (urban *M* = 6.4 versus rural *M* = 5.7, *p* = 0.05) and for practices in medical groups with a smaller proportion of Medicare patients (*p* = 0.01).

Table 1 displays the model-predicted practice leaders’ relative commitment scores for hypothetical practices with low, moderate, and high values for each implementation capability composite. For example, a practice with a low advanced access and tracking capabilities score (at the 25th percentile) would have a predicted practice leaders’ relative commitment score of 5.9, while a practice with a high score (75th percentile) would have a predicted practice leaders’ relative commitment score of 6.4.

**Table 1:** **Model-predicted practice leaders’ relative commitment to depression care improvement scores for practices with low, moderate, and high levels for each implementation capability composite**

	**Predicted relative commitment to improvement scores based on implementation capability composite scores**			***p***	***R*** ^**2**^
**Implementation capability composites**	**Low (25th percentile)**	**Moderate (50th percentile)**	**High** ^**a**^ **(75th percentile)**		
Depression collaborative care features in place	5.8	6.0	6.3	0.03	0.05
Advanced access and tracking capabilities	5.9	6.1	6.4	0.03	0.06
Depression culture and attitudes	5.4	6.1	6.5	<0.001	0.18
Prior depression quality improvement activities	5.4	6.1	6.8	<0.001	0.19
Quality improvement culture and attitudes	5.8	6.3	6.5	0.003	0.11

^a^The higher the value the greater the presence of facilitators and/or absence of barriers.

### Implementation wave

Of the 83 practices, ten were in wave 1 (Mar 2008); 20, in wave 2 (Sept 2008); 14, in wave 3 (Mar 2009); 11, in wave 4 (Sept 2009); 8 in, wave 5 (Mar 2010); and 20 ended up not implementing the DIAMOND care model (none). None of the practice or group demographic characteristics were associated with early initiation of implementation. As shown in Table 2, however, two of the proposed organizational readiness model elements (Figure 1) were significantly associated with implementation wave. These were practice leaders’ relative commitment (*p* = 0.002) and prior depression quality improvement activities (*p* = 0.03). Depression culture and attitudes showed a borderline significance (*p* = 0.05). Also as shown in Table 2, practice leaders’ relative commitment accounted for the greatest portion of the variance (*R*^2^ = 0.11), and for the two significant associations, the practice leaders’ relative commitment score was highest in wave 1 and the prior depression quality improvement activities score was highest in wave 1.

**Table 2:** **Values for practice leaders’ relative commitment to improvement scores and implementation capability composite scores based on timing of initiation of DIAMOND depression care improvement**

	**Timing of initiation: waves of practices initiating improvement**							
	**First**	**Second**	**Third**	**Fourth**	**Fifth**	**None**	***p*** ^**c**^	***R*** **2**
	**M (SD)**	**M (SD)**	**M (SD)**	**M (SD)**	**M (SD)**	**M (SD)**		
Practice leaders’ relative commitment scores^a^	8.0	6.8	5.7	5.8	4.6	5.7	0.002	0.11
	(0.78)	(1.0)	(1.6)	(1.5)	(2.1)	(1.6)		
Depression collaborative care features in place^b^	0.33	0.25	0.26	0.28	0.13	0.24	0.30	0.01
	(0.15)	(0.13)	(0.20)	(0.17)	(0.11)	(0.16)		
Advanced access and tracking capabilities^b^	0.58	0.59	0.58	0.60	0.44	0.58	0.69	0.00
	(0.16)	(0.15)	(0.19)	(0.14)	(0.15)	(0.15)		
Depression culture and attitudes^b^	0.79	0.73	0.76	0.62	0.60	0.66	0.05	0.05
	(0.12)	(0.16)	(0.15)	(0.20)	(0.23)	(0.21)		
Prior depression quality improvement activities^b^	0.50	0.28	0.37	0.34	0.15	0.28	0.03	0.06
	(0.14)	(0.14)	(0.20)	(0.18)	(0.13)	(0.16)		
Quality improvement culture and attitudes^b^	0.76	0.76	0.73	0.72	0.63	0.74	0.58	0.00
	(0.09)	(0.12)	(0.17)	(0.19)	(0.26)	(0.13)		

^a^Values for practice leaders’ relative commitment to improvement scores (higher practice leaders’ relative commitment scores reflect higher relative commitment).

^b^Implementation capability composite scores (higher implementation capability scores reflect greater presence of facilitators and/or absence of barriers).

^c^ *p* values and *R* ^2^ values are for the regression coefficients from a general linear regression model predicting wave from each of the relative commitment and implementation capability composites.

## Discussion

Studies of depression care improvement in primary care only infrequently account for baseline differences between practices in organizational readiness. Yet knowledge of readiness could provide opportunities for more effective intervention designs and more informative evaluation results. This study took advantage of the opportunity to explore organizational readiness in 83 primary care practices studied in the DIAMOND initiative for implementing depression collaborative care. Study results provide empirical support for several key organizational readiness concepts within the Weiner theoretical framework [30] and, in doing so, provide a basis for further investigation of this critical but often ignored determinant of implementation success. In addition, study results suggest the potential usefulness and feasibility of practice leader surveys for assessing readiness, including use of a single question on relative commitment to depression care improvement as well as more detailed assessments of specific improvement capabilities.

Weiner’s theoretical framework, while derived from extensive review of prior studies [13], has undergone little empirical testing. The framework highlights psychological readiness based on change commitment and change efficacy (i.e., the degree to which an improvement community such as a practice collectively values a proposed change, commits to and feels favorable and confident about implementation). It also postulates that implementation capability, meaning the concrete task demands, resource availability, and situational factors relevant to the specific improvements to be undertaken, strongly influences change efficacy. The survey items used in this study were previously tested [26,28] and were derived, in part, from sources such as the National Committee for Quality Assurance. The items were originally designed to reflect elements of the Chronic Illness Care model [2,3,28]. We used them to develop measures that would mirror the concepts of change commitment and implementation capability (Figure 1). Our measure of practice leaders’ relative commitment (reflecting change commitment) is an attitudinal measure of psychological readiness as specified in the Weiner model. Our measures of implementation capability reflect barriers and facilitators to depression collaborative care. And, consistent with the Weiner model, we found a significant relationship between practice leaders’ relative commitment to depression care improvement and our measures of implementation capability.

There are some differences between our survey items and the specifics of the Weiner model. In measuring change commitment (relative commitment), we surveyed practice leaders rather than obtaining a wider representation of the entire practice. However, given the extensive literature on the role of leadership support for improvement [31], the likelihood that a practice will actually undertake a major improvement if leaders do not prioritize it is markedly reduced. We also think that onsite leaders both shape and reflect the values and culture of their practices. Based on these assumptions, assessing practice clinical and quality leaders’ relative commitment may be a valuable and feasible alternative to surveying all practice members.

In Weiner’s theory, initiation of implementation is one of the outcomes of organizational readiness. As an exploratory finding, our data suggest that practice leaders’ relative commitment and prior depression quality improvement activities may have predicted which practices would initiate the DIAMOND implementation early (e.g., early adopters [29]). This finding provides support for further development of the concept of organizational stages of change [23] as illustrated in Figure 1. Viewing organizational readiness as developing in progressive stages would have important implications for depression collaborative care intervention and evaluation.

Although all implementation capability composites we tested were independently significantly associated with relative commitment, and contributed explanatory power for predicting it, the associations for depression culture and attitudes and for prior depression quality improvement activities showed the most robust statistical significance. These findings show the potential value of considering improvement specific (in this case, depression-specific) capabilities separately from more general capabilities in assessing and addressing readiness. Future research should determine, for example, whether a different approach to improvement (such as an emphasis on mental health needs assessment and education) is needed for practices that have not developed a culture favorable to depression care improvement.

Overall, improving the match between the types of assistance offered by initiative proponents and the needs of the participating organizations and practices might reduce variations across practices in the success of depression improvement initiatives [15,18,21,27,30]. The constructs we identified could become part of the initial multiphasic practice assessments or quality improvement prework used by a variety of quality improvement methodologies [4,29].

Practice leaders’ relative commitment to depression care improvement was stronger than any of the individual implementation capability composites in predicting participation in the early implementation wave. These data suggest that practice leaders may integrate a wide range of knowledge about local conditions relevant to implementation challenges when expressing a relative commitment to improvement. Information on the relative commitment of practice leaders to depression care improvement could help to efficiently identify potential early adopters of depression care improvement. Diffusion theory [29,32] predicts that early adopters (i.e., individuals or organizations ready and willing to undertake an improvement) pave the way for those with less enthusiasm or capability.

For researchers carrying out quality improvement studies, it is often challenging to match sites or adjust results based on site-level organizational characteristics. A valid summary measure of readiness could assist evaluations of multi-practice implementation efforts by adjusting expectations for improvement at the practice level. While this study is preliminary in this regard, the measures we used may hold promise for practice matching and subgroup analysis of study results. While the individual relationships between the implementation capability composites and relative commitment remain valid, our results on correlations show that the composites are not empirically distinct. Further research on the composition of the five implementation capability composites to improve ease of use and maximize structural and discriminant validity (e.g., a systematic approach to shortening the number and determining the unidimensionality of the items) may be warranted. We looked at the effect of data reduction on the scales used here by sequentially removing items with a standardized variable correlation of less than 0.300 and 0.200 but found little change in the overall score or Cronbach’s alpha and did not remove any items.

Our findings suggest that practice leaders located in urban areas or belonging to medical groups with a lower proportion of Medicare patients were more likely to indicate a high relative commitment to depression care improvement. Greater exposure to trends and subsequent demand for services by patients may explain the higher relative commitment expressed in urban practices; whereas, limited reimbursement for depression care and/or different attitudes to depression in the elderly may explain lower relative commitment ratings in groups with more Medicare patients.

The data and analyses we present are exploratory. Several aspects of the study present limitations. The observational and cross-sectional nature of the study precludes assessment of causation. Our sample size, while substantial for a practice-level study, is limited for statistical analyses. Practice leaders’ relative commitment to improvement and their perception of implementation capability reflect only practice leadership reports; assessment of all site clinicians and staff may have provided additional or different information. The collinearity of the tested implementation capability composites would affect the relative significance and variance of the individual composites if tested together in relationship to relative commitment. Timing of initiation as measured by implementation wave is an exploratory variable that reflects both study management as well as practice level influences.

The large number of practices surveyed, participation by multiple medical groups, the gathering of independent responses from clinical and quality leaders of the practices, the high response rate, and timing of the measures of commitment and capability prior to program implementation are strengths of the study. The use of an *a priori* conceptual model is an additional strength. Our analyses provide preliminary empirical confirmation of Weiner’s framework of organizational readiness, and therefore, a strong basis for further trialing and investigation of the framework and for further development of usable tools for depression quality improvement initiatives and studies.

## Conclusions

This study provides preliminary evidence suggesting that the measures of practice leaders’ relative commitment and organizational implementation capability (consistent with Weiner’s theoretical model of organizational readiness) are significantly interrelated. Either or both of these measures may be useful in understanding and reducing variations in implementation success for depression care improvement initiatives. Future studies of organizational readiness may further refine the concepts and their measures for depression, and may explore use of similar approaches for other types of improvements.

## Additional file

Additional file 1:
**Items from the PPC and CPCQ grouped into categories.** Items from the Physician Practice Connection Questionnaire and the Change Process Capability Questionnaire* grouped in categories to assess implementation capability and to predict relative commitment to depression care improvement.

## Abbreviations

DIAMOND: Depression Improvement Across Minnesota, Offering a New Direction
EMR: electronic medical record
GLM: general linear regression model
ICSI: Institute for Clinical Systems Improvement
M: mean
MD: medical doctor
SD: standard deviation
