# Supplementary material for: Assessing organizational readiness for depression care quality improvement: relative commitment and implementation capability
Source: Implement Sci. 2014 Dec 2;9:173. doi: 10.1186/s13012-014-0173-1 (PMC4276014; doi:10.1186/s13012-014-0173-1)
Supplement: Additional file 1: — Items from the PPC and CPCQ grouped into categories. Items from the Physician Practice Connection Questionnaire and the Change Process Capability Questionnaire* grouped in categories to assess implementation capability and to predict relative commitment to depression care improvement. [file 13012_2014_173_MOESM1_ESM.docx]

**Additional file 1. Items from the Physician Practice Connection Questionnaire (PPC) and the Change Process Capability Questionnaire (CPCQ)* Grouped in Categories to Assess Relative Commitment to Depression Care Improvement**

| **Categories** | **Items** |
| --- | --- |
| Advanced Access and Tracking Capabilities  (18 items) | - Does your clinic(s) have the following standardized processes to support patient access and communication with the clinic(s)?, PPC   - scheduling patients with a personal clinician whenever possible *(Yes, works well; Yes, needs improvement; No; Don't know)*   - scheduling same day appointments based on patient's requests *(Yes, works well; Yes, needs improvement; No; Don't know)* - Does your clinic(s) use the following electronic or paper-based charting tools to organize and document clinical information in the medical   record?:, PPC   - - problem list *(Yes, works well; Yes, needs improvement; No; Don't know)*   - flow sheet for depression care *(Yes, works well; Yes, needs improvement; No; Don't know)*   - list of over-the-counter medications, supplements, and alternative therapies *(Yes, works well; Yes, needs improvement; No; Don't know)*   - list of prescribed medications, including both chronic and short-term *(Yes, works well; Yes, needs improvement; No; Don't know)*   - a place to record at least 3 age-appropriate risk factors *(Yes, works well; Yes, needs improvement; No; Don't know)*   - a place to record age-appropriate preventive services *(Yes, works well; Yes, needs improvement; No; Don't know)* - Does your clinic(s) have a system to identify and send reminders to patients who are due for the following services?, PPC   - a follow-up visit *(Yes, works well; Yes, needs improvement; No; Don't know)*   - prescription renewal for antidepressants *(Yes, works well; Yes, needs improvement; No; Don't know)*   - preventive services (e.g., immunizations, mammograms) *(Yes, works well; Yes, needs improvement; No; Don't know)* - Does your clinic(s) use an electronic data system that enables identification of the status of age-appropriate preventive services for your clinic as a whole?, PPC *(Yes, works well; Yes, needs improvement; No; Don't know)* - What best described the medical records and information systems at your practice site?, PPC (Paper record only; Paper record+; EMR--separate ordering data systems; EMR--all functions; other) - For each of the following conditions or preventive services, does your clinic(s) use guideline-based reminders, flowsheets, checklists, or other templates to remind physicians about needed services at the point of seeing the patient? These can include alerts within an EMR or paper notes attached to the front of the chart?, PPC   - depression *(Yes, works well; Yes, needs improvement; No; Don't know)*   - age-appropriate screening tests *(Yes, works well; Yes, needs improvement; No; Don't know)*   - age appropriate immunizations *(Yes, works well; Yes, needs improvement; No; Don't know)*   - age-appropriate risk assessments *(Yes, works well; Yes, needs improvement; No; Don't know)*   - counseling *(Yes, works well; Yes, needs improvement; No; Don't know)* |
| Depression Collaborative Care Features in Place  (29 items) | - Do the non-physician members of your staff share responsibility for managing care for patients with depression in the following ways?, PPC   - executing standing orders (e.g., medication refills, test orders, delivery of routine preventive services) *(Yes, works well; Yes, needs improvement; No; Don't know)*   - coordinating depression care with external disease management or case management organizations *(Yes, works well; Yes, needs improvement; No; Don't know)* - What components of care management are routinely provided to your patients with depression?, PPC   - Perform pre-visit planning to assure that all needed information is available at the time of the visit (e.g., consult reports, PHQ9 scores) *(Yes, works well; Yes, needs improvement; No; Don't know)*   - measure depression severity at least monthly for newly treated patients*(Yes, works well; Yes, needs improvement; No; Don't know)*   - evaluate each patient newly diagnosed with depression for alcohol misuse and chemical dependency *(Yes, works well; Yes, needs improvement; No; Don't know)*   - identify and review all prescribed and OTC medications, supplements, and alternative therapies with patients at each visit *(Yes, works well; Yes, needs improvement; No; Don't know)*   - complete after-visit follow-up for depression care (e.g., by a nurse or care manager) after each visit *(Yes, works well; Yes, needs improvement; No; Don't know)*   - closely monitor patient response and adherence to the care plan for depression *(Yes, works well; Yes, needs improvement; No; Don't know)*   - follow-up when patients with depression have not kept important appointments *(Yes, works well; Yes, needs improvement; No; Don't know)* - Does your clinic(s) have a system in place to assure that patients with depression, PPC   - Receive more specific diagnostic codes than 311 (e.g., 296.2, 296.3) *(Yes, works well; Yes, needs improvement; No; Don't know)*   - Receive treatment intensification if they fail to improve *(Yes, works well; Yes, needs improvement; No; Don't know)*   - Are routinely reviewed by a psychiatrist if they are complex or failing to improve *(Yes, works well; Yes, needs improvement; No; Don't know)* - What components of care management are routinely provided to your patients with depression?, PPC   - review and individualize the care management plan with patients *(Yes, works well; Yes, needs improvement; No; Don't know)*   - help patients set individualized treatment goals *(Yes, works well; Yes, needs improvement; No; Don't know)*   - review patients' history of targeted clinical measurements over time (eg. {PHQ9 scores) *(Yes, works well; Yes, needs improvement; No; Don't know)*   - assess barriers when patients have not met treatment goals*(Yes, works well; Yes, needs improvement; No; Don't know)*   - assess barriers when patients have not filled, refilled, or taken prescribed medications*(Yes, works well; Yes, needs improvement; No; Don't know)*   - provide a personalized relapse prevention plan to patients finishing active treatment*(Yes, works well; Yes, needs improvement; No; Don't know)* - Are the following care management services in place to proactively identify and assist patients who receive depression care in inpatient or emergency facilities? These can be performed by the clinic(s) or an external organization: , PPC   - identification of patients who have received depression care elsewhere (including hospitals, skilled nursing facilites, or ERs) *(Yes, works well; Yes, needs improvement; No; Don't know)*   - follow-up calls to patients with depression after discharge form other facilities *(Yes, works well; Yes, needs improvement; No; Don't know)*   - coordination of care with external disease management or case management organizations, as appropriat*(Yes, works well; Yes, needs improvement; No; Don't know)*e - Check the appropriate boxes if you have a nurse or comparable person who serves as a care manager (provides education, follow-up-, etc.) for patients with each of the following conditions, PPC (Depression, diabetes, heart disease, asthma, none) - Do the non-physician members of your staff share responsibility for managing care for patients with depression in the following ways?, PPC   - reminding patients of appointments *(Yes, works well; Yes, needs improvement; No; Don't know)*   - educating patients about self-care for depression *(Yes, works well; Yes, needs improvement; No; Don't know)* - For patients with depression, does your clinic(s) routinely use the following activities to encourage patient self-management?, PPC   - provide or connect patients to self-management support programs *(Yes, works well; Yes, needs improvement; No; Don't know)*   - provide educational resources in the language or medium that the patient understands*(Yes, works well; Yes, needs improvement; No; Don't know)*   - assess patient preferences, readiness to change, and self-management abilities *(Yes, works well; Yes, needs improvement; No; Don't know)* - Strategies to implement improved depression care: Delegating to non-physician staff the responsibility to carry out aspects of depression care that are normally the responsibility of physicians, CPCQ (Yes, works well; Yes, but did not work well; No, not used) - Do the non-physician members of your staff share responsibility for managing care for patients with depression in the following ways?, PPC   - following patients treated for depression to assure guideline care *(Yes, works well; Yes, needs improvement; No; Don't know)* |
| Depression Culture and Attitudes  (5 items) | - Have all the clinicians in your practice agreed to follow evidence-based treatment guidelines for the conditions and preventive services—depression?, PPC (Yes, No, Don't know) - Clinicians in our medical group believe that good depression care is very important, CPCQ (Strongly disagree, Somewhat disagree, Neither agree nor disagree, Somewhat agree, Strongly agree) - Our resources (personnel, time, financial) are too tightly limited to improve depression care, CPCQ (Strongly disagree, Somewhat disagree, Neither agree nor disagree, Somewhat agree, Strongly agree) - Our medical group leadership is strongly committed to the need for change in depression care and for leading that change, CPCQ (Strongly disagree, Somewhat disagree, Neither agree nor disagree, Somewhat agree, Strongly agree) - The clinicians in our medical group are very interested in improving depression care, CPCQ (Strongly disagree, Somewhat disagree, Neither agree nor disagree, Somewhat agree, Strongly agree) |
| Prior Depression Quality Improvement Activities  (25 items) | - Strategies to implement improved depression care: , CPCQ   - Providing information and skills-training related to improved depression care (Yes, works well; Yes, but did not work well; No, not used)   - Use of opinion leaders, role modeling or other vehicles to encourage support for changes in depression care (Yes, works well; Yes, but did not work well; No, not used)   - Changing or creating systems in the medical group that make it easier to provide good depression care (Yes, works well; Yes, but did not work well; No, not used)   - Removal or reduction of barriers to better depression care (Yes, works well; Yes, but did not work well; No, not used)   - Organizing people into teams focused on accomplishing the change process for depression care (Yes, works well; Yes, but did not work well; No, not used)   - Using periodic measurement of depression care for the purpose of assessing compliance with the new approach to depression care (Yes, works well; Yes, but did not work well; No, not used)   - Reporting measurements of individual or care unit performance for depression care by comparison with their peers (Yes, works well; Yes, but did not work well; No, not used)   - Setting goals and benchmarking rates of depression care at least yearly (Yes, works well; Yes, but did not work well; No, not used)   - Customizing the implementation of depression care changes to each site of care (Yes, works well; Yes, but did not work well; No, not used)   - Use of rapid cycling, piloting, pre-testing, or other vehicles for reducing the risk of negative results from introducing organization-wide change in depression care (Yes, works well; Yes, but did not work well; No, not used)   - Deliberately designing depression care improvements so as to make physician participation less work than before (Yes, works well; Yes, but did not work well; No, not used)   - Deliberately designing depression care as a means to make the care more beneficial to the patient (Yes, works well; Yes, but did not work well; No, not used) - Does your clinic(s) measure or receive results on the following types of performance measure for depression care?, PPC   - clinical process (e.g., % of depressed patients who continue on antidepressants for 3 to 6 months) *(Yes, works well; Yes, needs improvement; No; Don't know)*   - clinical outcomes (e.g., depression response or remission rates) *(Yes, works well; Yes, needs improvement; No; Don't know)*   - patient experience of care from surveys of patients *(Yes, works well; Yes, needs improvement; No; Don't know)* - Do your quality improvement activities for depression care include: , PPC   - setting goals based on measurement results *(Yes, works well; Yes, needs improvement; No; Don't know)*   - taking action to improve performance of individuals physicians *(Yes, works well; Yes, needs improvement; No; Don't know)*   - taking action to improve performance at the clinic(s) as a whole *(Yes, works well; Yes, needs improvement; No; Don't know)* - We have greatly improved the process of depression care in the past year, CPCQ (Strongly disagree, Somewhat disagree, Neither agree nor disagree, Somewhat agree, Strongly agree) - We have clinical champions interested in leading the improvement of depression care, CPCQ (Strongly disagree, Somewhat disagree, Neither agree nor disagree, Somewhat agree, Strongly agree) - The leaders of our efforts to improve depression care are enthusiastic about their task, CPCQ (Strongly disagree, Somewhat disagree, Neither agree nor disagree, Somewhat agree, Strongly agree) - The new process of care for depression is more advantageous than the old to everyone involved (patients, personnel, and medical group), CPCQ (Strongly disagree, Somewhat disagree, Neither agree nor disagree, Somewhat agree, Strongly agree) - Strategies to implement improved depression care: Providing to those who are charged with implementing improved depression care the power to authorize and make the desired changes, CPCQ - Does your clinic(s) have a system outside of the paper chart to track care for patients with depression?, PPC   - registry of all patients being treated for depression to monitor programs and track follow-up needs *(Yes, works well; Yes, needs improvement; No; Don't know)*   - tracking of mental health referrals until the consult report returns *(Yes, works well; Yes, needs improvement; No; Don't know)* |
| Quality Improvement Culture and Attitudes  (14 items) | - Our medical group clinic operations rely heavily on organized systems, CPCQ (Strongly disagree, Somewhat disagree, Neither agree nor disagree, Somewhat agree, Strongly agree) - Our medical group has well-developed administrative structures and processes in place to create change, CPCQ (Strongly disagree, Somewhat disagree, Neither agree nor disagree, Somewhat agree, Strongly agree) - Our medical group has a well-defined quality improvement process for designing and introducing changes in the quality of care, CPCQ (Strongly disagree, Somewhat disagree, Neither agree nor disagree, Somewhat agree, Strongly agree) - Have all the clinicians in your practice agreed to follow evidence-based treatment guidelines for conditions and preventive services, PPC   - Age-appropriate screening tests (Yes, No, Don't know)   - Age-appropriate immunizations (Yes, No, Don't know)   - Age-appropriate risk assessments (Yes, No, Don't know)   - Counseling (Yes, No, Don't know) - The thinking of our leadership is strongly oriented toward systems, CPCQ (Strongly disagree, Somewhat disagree, Neither agree nor disagree, Somewhat agree, Strongly agree) - Our medical group attaches more priority to quality of care than to finance, CPCQ (Strongly disagree, Somewhat disagree, Neither agree nor disagree, Somewhat agree, Strongly agree) - The clinicians in our medical group espouse a shared mission, CPCQ (Strongly disagree, Somewhat disagree, Neither agree nor disagree, Somewhat agree, Strongly agree) - The clinicians in our medical group adhere to medical group policies, CPCQ (Strongly disagree, Somewhat disagree, Neither agree nor disagree, Somewhat agree, Strongly agree) - Our medical group is undergoing considerable stress as the result of internal changes, CPCQ (Strongly disagree, Somewhat disagree, Neither agree nor disagree, Somewhat agree, Strongly agree) - The working environment in our medical group is collaborative and cohesive, with shared sense of purpose, cooperation, and willingness to contribute to the common good, CPCQ (Strongly disagree, Somewhat disagree, Neither agree nor disagree, Somewhat agree, Strongly agree) - Our medical group understands and uses quality improvement skills effectively, CPCQ (Strongly disagree, Somewhat disagree, Neither agree nor disagree, Somewhat agree, Strongly agree) |

*The Physician Practice Connection Questionnaire was developed by the NCQA (National Committee for Quality Assurance) to measure practice system implementation of the Chronic Care Model. The Change Process Capability Questionnaire was developed by Dr. Leif Solberg to measure organizational factors and strategies identified as relevant by a panel of physician leaders and quality improvement coordinators with expertise in successful implementation of medical practice guidelines.
